# Elevated temperatures increase abnormalities in embryos and reduce larval survival in serpulid polychaetes

**DOI:** 10.1242/bio.060053

**Published:** 2023-09-13

**Authors:** J. Pablo Sánchez-Ovando, Francisco Benítez-Villalobos, J. Rolando Bastida-Zavala

**Affiliations:** ^1^ Laboratorio de Sistemática de Invertebrados Marinos (LABSIM), Instituto de Recursos, Universidad del Mar (UMAR), campus Puerto Ángel, Ciudad Universitaria, Oaxaca 70902, México; ^2^ Laboratorio de Ecología del Desarrollo (ECODES), Instituto de Recursos, Universidad del Mar (UMAR), campus Puerto Ángel, Ciudad Universitaria, Oaxaca 70902, México

**Keywords:** Critical temperature, Larvae, *Spirobranchus*, Thermal tolerance, Trochophore

## Abstract

Environmental temperature is one of the most significant factors influencing the developmental rate and survival of embryos and larvae of many marine animals, including polychaetes. The aim of this study was to experimentally evaluate the effect of temperature increase on the embryonic development and larval survival of *Spirobranchus incrassatus* and *S.* cf. *corniculatus*. Adult worms of both species were collected from the western margin of the Gulf of Tehuantepec, Mexico. Embryos and larvae were obtained from these worms and exposed to four temperature treatments (28, 30, 32 and 34°C). The optimal temperature for embryonic development of *S. incrassatus* and *S.* cf. *corniculatus* was 30 and 28°C, respectively. For both species, the maximum critical temperature was 32°C and the lethal temperature was 34°C. The embryonic stages of *S.* cf. *corniculatus* were most negatively affected by elevated temperatures. Larval survivorship of *S. incrassatus* and *S.* cf. *corniculatus* was higher at 30°C and 28°C (76.2±2.8%; 72.6±4.2%) and lower at 34°C (28.9±2.6%; 26.3±2.9%), respectively. These results suggest that *S.* cf. *corniculatus* has the lowest thermal tolerance. Both *Spirobranchus* species already live near their upper limit of thermal tolerance in the study region (30°C). In the near future, under a global warming scenario, the distribution of both species could be modified, causing a loss of biodiversity, changes in the trophic chain, and alterations in the water column, such as excess organic matter.

## Teaser

**Summary:** This work describes the effect of increased temperature on the early developmental stages of two serpulid polychaetes. We demonstrated that increased temperatures induced embryo abnormalities and decreased larval survival.

## INTRODUCTION

In marine ecosystems, many environmental factors, such as temperature, salinity, pH, and nutrient availability affect the presence, distribution, and development of marine animals. However, numerous studies have indicated that temperature is one of the main factors that determine the survival of species and influences their physiological processes (Levitan, 1995; Kupriyanova et al., 2001; Sanford, 2002; O'Connor et al., 2007; Nguyen et al., 2011).

Marine invertebrates are vulnerable to temperature changes during early development. An increase in temperature above the normal range can cause abnormalities in embryonic and larval development. For instance, in some invertebrates such as echinoderms, polychaetes, and crustaceans, larvae have been described as malformed, failing to settle, or dying (Thorson, 1950; Rupp, 1973; Crisp, 1977; Bartolini et al., 2013; Lamare et al., 2014; Mejía-Gutiérrez et al., 2019).

The Serpulidae (Rafinesque) family is one of the most abundant and well-known families of polychaetes, comprising 506-576 species and 74 accepted genera (Bastida-Zavala and Sánchez-Ovando, 2021; Rouse et al., 2022). All the serpulids are suspension-feeding sessile tubeworms, build their tubes with CaCO_3_, and attach to several types of hard substrates (Bastida-Zavala and Sánchez-Ovando, 2021). Therefore, they are ecologically important because they feed on particles suspended in the water column (Jordana et al., 2000; Ten Hove and Kupriyanova, 2009; Kupriyanova et al., 2019). In particular, species of the genus *Spirobranchus* (Blainville), play a key role in marine environments because some species are gregarious and form large structures known as serpulid reefs, which serve as a habitat and refuge for other marine invertebrate species (Gosselin and Sewell, 2012; Pazoki et al., 2020; Sivananthan et al., 2021; Nishi et al., 2022).

Three species of *Spirobranchus* are distributed in the eastern tropical Pacific: *Spirobranchus incrassatus* (Krøyer [in] Mörch), *Spirobranchus minutus* (Rioja), and *Spirobranchus* cf. *corniculatus* (Bastida-Zavala, 2008, as *Spirobranchus* cf. *gaymardi*), but *S. incrassatus* and *S.* cf. *corniculatus* are the largest (0.8-5 cm and 1.5-11.8 cm, respectively) and therefore the easiest to collect and handle. *Spirobranchus incrassatus* is distributed along the Pacific coast from the southern Gulf of California to Colombia and the Galapagos Islands (Keppel et al., 2019). *Spirobranchus* cf. *corniculatus* has a somewhat more limited distribution from Baja California to Oaxaca, Mexico (Bastida-Zavala, 2008). Both species can be found in the Gulf of Tehuantepec, Mexico, where the average sea surface temperature is 28.5°C because this area is located within the eastern Pacific warm pool (Wang and Enfield, 2001; Misra et al., 2016). In winter, the coastal dynamics are strongly influenced by a series of northerly wind events known as ‘Nortes’ or ‘Tehuanos’. During ‘Tehuanos’ events in the central region of the Gulf of Tehuantepec the temperature can drop to 23-25°C while at the edges the temperature is around 30°C (Lavín et al., 1992). Other authors have indicated that the sea surface temperature varies from 26-27°C during the winter to 28.5-29.5°C in summer (Wilkinson et al., 2009). Due to the strong variability of oceanic and atmospheric conditions present in the Gulf of Tehuantepec, this region might be considered as a natural laboratory for investigating the effect of future temperature increase. Recently, Sánchez-Ovando et al. (2021) described the early development of these two *Spirobranchus* species.

In serpulids, the effect of temperature has been investigated at some stages of the life cycle, such as gametes, embryos, larvae, and juveniles. For example, Vuillemin (1958) indicated that the larval development time of the exotic serpulid, *Ficopomatus enigmaticus* (Fauvel), increased as the temperature decreased, 7 h at 30°C and 15 h at 20°C. Leone (1970) indicated that the number of gametes and the maturation of *Hydroides dianthus* (Verrill) decreased at high temperatures (26 and 30°C). Kupriyanova and Havenhand (2005) found that low (11-16°C) and high (26-31°C) temperatures reduced fertilization success and the duration of sperm activity in *Galeolaria caespitosa* (Lamarck). Gee (1967) observed that at low temperatures (5°C) the maturation of *Spirorbis* (*Spirorbis*) *rupestris* (Gee & Knight-Jones) gametes is slow; while at high temperatures (10, 15 and 20°C) maturation is faster. On the other hand, some species of serpulids, such as *Hydroides elegans* (Haswell), are tolerant of high temperature variations. (Qiu and Qian, 1997, 1998). Specifically, the effect of temperature on *Spirobranchus* has been investigated in a few species such as *Spirobranchus sinuspersicus*, *Spirobranchus cariniferus* and *Spirobranchus bakau*. Negative effects on embryonic and larval development have been described in these species, causing the death of larvae and poor settlement success (Crisp, 1977; Castric-Fey, 1984; Lavajoo and Amrollahi-Biuki, 2015; Gosselin et al., 2019).

All *Spirobranchus* species studied thus far are broadcast spawners with subsequent development in the water column until the larvae settle on the seabed and metamorphose to develop into juveniles (Kupriyanova et al., 2001). Temperature controls many of these life cycle stages, and it is known that the effect of temperature on serpulid species depends on their thermal tolerance. Therefore, this abiotic environmental factor can be a serious threat to the fitness, adaptability, and persistence of offspring of many marine invertebrate species, including serpulids (Byrne, 2012; Byrne and Przeslawski, 2013).

*Spirobranchus incrassatus* and *S.* cf. *corniculatus* have a key ecological role in the marine environment where they are distributed. Despite the importance of these two serpulid species, no research has been done to determine how they will be affected by expected temperature increases in the near future. For this reason, the aim of this study was to evaluate experimentally the effect of temperature increase (28, 30, 32, and 34°C) on the embryonic development and larval survival of these two *Spirobranchus* species to determine the thermal tolerance of both species during the early stages of development.

## RESULTS

### Effect of temperature on the embryonic development of *S. incrassatus* and *S.* cf. *corniculatus*

*Spirobranchus incrassatus* and *S.* cf. *corniculatus* embryos cultured at 28°C developed into early trochophore larvae at 12 and 16 h post-fertilization, respectively (Figs 2A and 3A). The larvae of both species developed at a faster rate (8 and 12 h post-fertilization, respectively) at 30°C (Figs 2B and 3B).

**Fig. 1.**
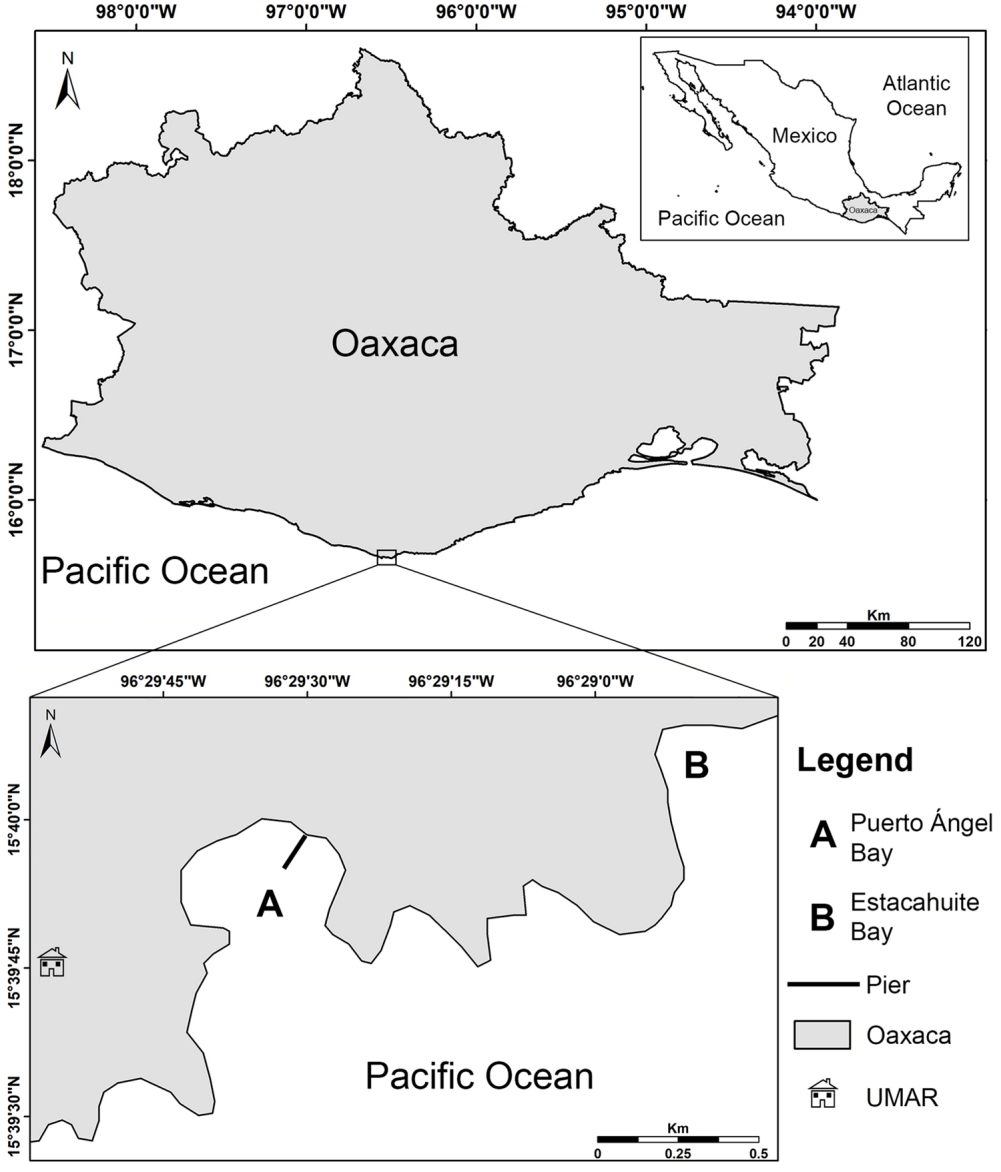
**Study area.** (A) Puerto Ángel Harbor and (B) Estacahuite Bay, southern Mexican Pacific.

**Fig. 2.**
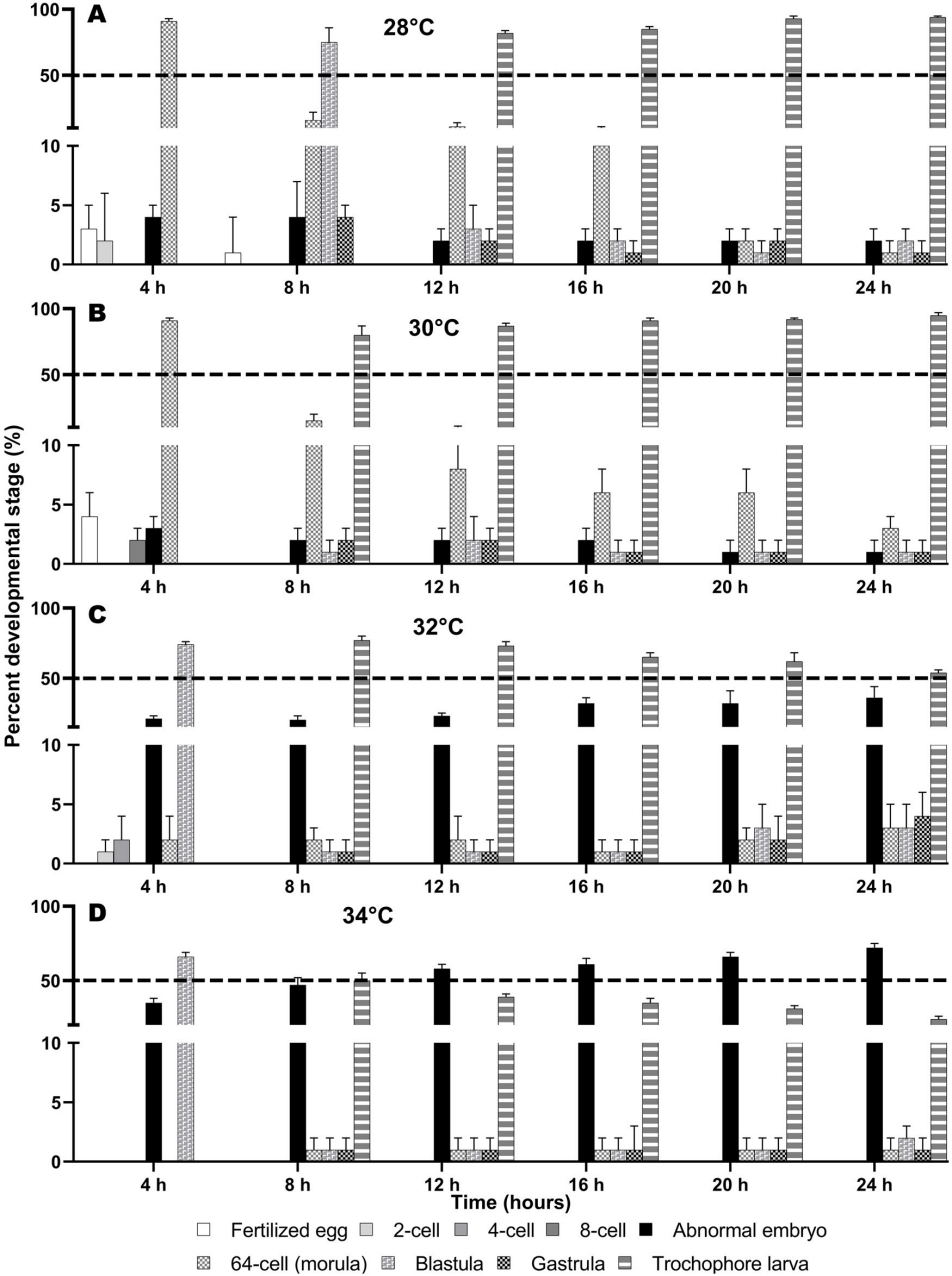
**Effect of temperature on the embryonic development of *S. incrassatus*.** (A-D) Percentage (mean±SD, *n*=6) of developmental stages achieved at every time point during the experimental period (24 h) at the four temperature treatments.

**Fig. 3.**
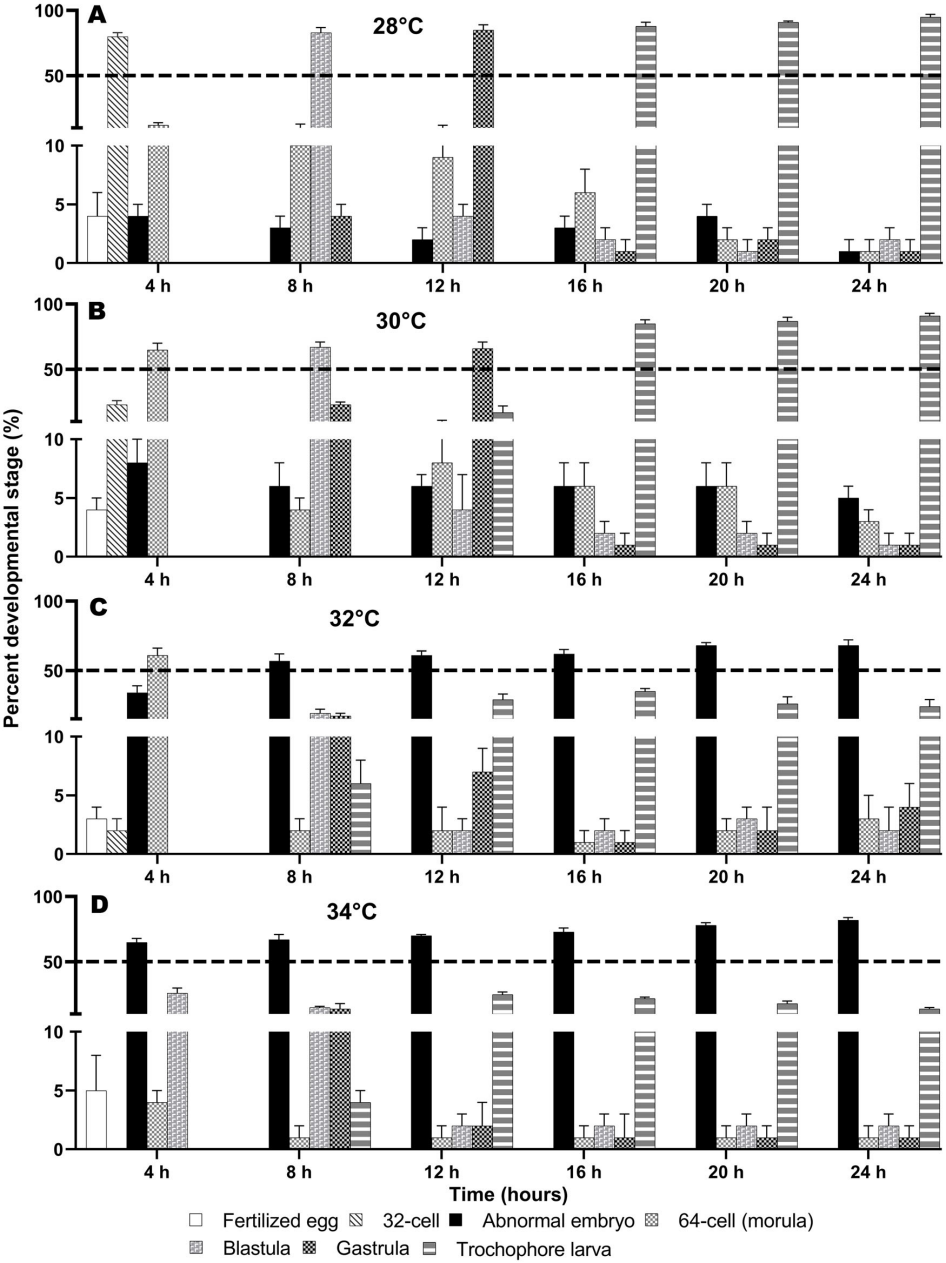
**Effect of temperature on the embryonic development of *S.* cf. *corniculatus*.** (A-D) Percentage (mean±SD, *n*=6) of developmental stages achieved at every time point during the experimental period (24 h) at the four temperature treatments.

For *S. incrassatus*, a high percentage of normal embryos (>50%) were obtained at all sampling times (4, 8, 12, 16, 20, and 24 h) at 28, 30, and 32°C; however, at the extremely high temperature, there was an increase in abnormal embryos (21-36%). At 34°C, abnormal embryos were predominant after 12 h (58-72%) (Table 1, Fig. 2A-D). Regarding *S.* cf. *corniculatus*, at all sampling times a high percentage of normal embryos (> 50%) were obtained at 28 and 30°C; whereas at 32°C, the percentage of abnormal embryos increased (57-68%) after 8 h and at 34°C the percentage of abnormal embryos increased significantly (65-82%) at all sampling times (Table 2, Fig. 3A-D). The ANOSIM indicated that in both species there were statistically significant differences in the percentage of embryos of each stage found between temperatures (*S. incrassatus*: *P*<0.001; *S.* cf. *corniculatus*: *P*<0.001), while between experiments (E1 and E2) there were no significant differences (*S. incrassatus*: *P*=0.812; *S.* cf. *corniculatus*: *P*=0.999).

**Table 1.**
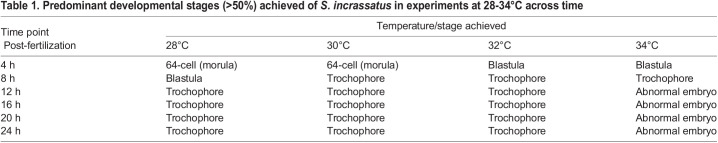
Predominant developmental stages (>50%) achieved of *S. incrassatus* in experiments at 28-34°C across time

**Table 2.**
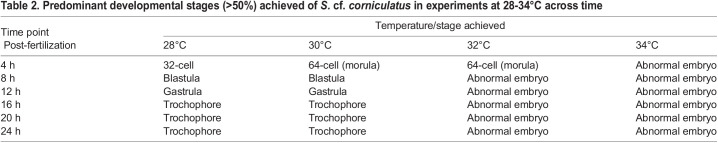
Predominant developmental stages (>50%) achieved of *S.* cf. *corniculatus* in experiments at 28-34°C across time

**Fig. 4.**
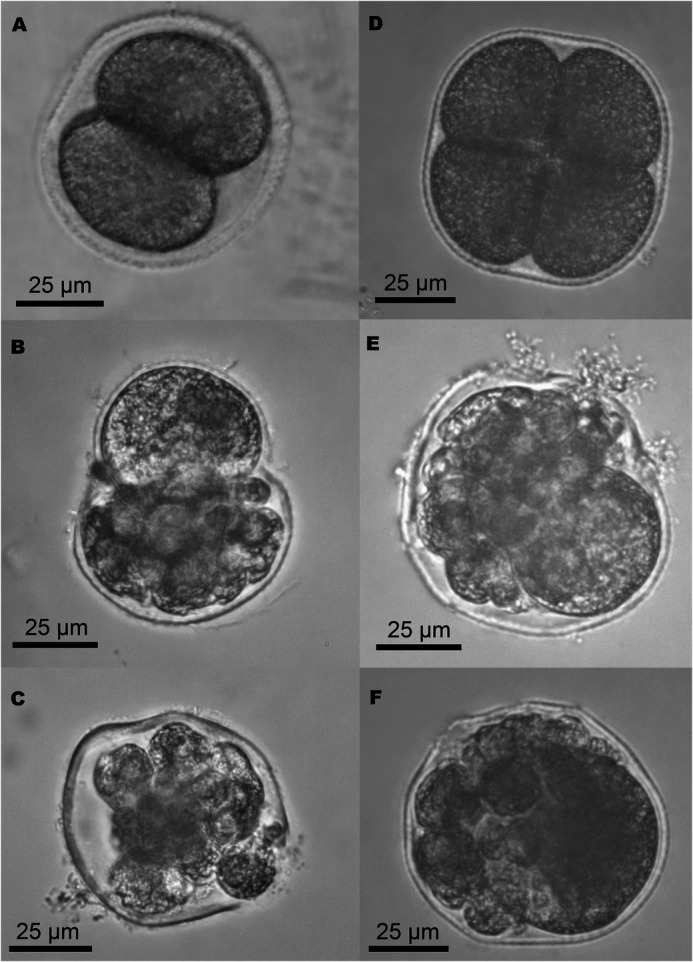
**Normal and abnormal embryos of the two *Spirobranchus* species.** (A) Normal embryo of *S. incrassatus* in the first cell division with two blastomeres. (B,C) Abnormal embryos of *S. incrassatus*. (D) Normal embryo of *S.* cf. *corniculatus* in the second cell division with four blastomeres. (E,F) Abnormal embryos of *S.* cf. *corniculatus*.

Unlike normal embryos (Fig. 4A,D), abnormal embryos were characterized by incomplete cleavage with blastomeres of different sizes or broken egg envelopes (Fig. 4B,C,E,F).

**Fig. 5.**
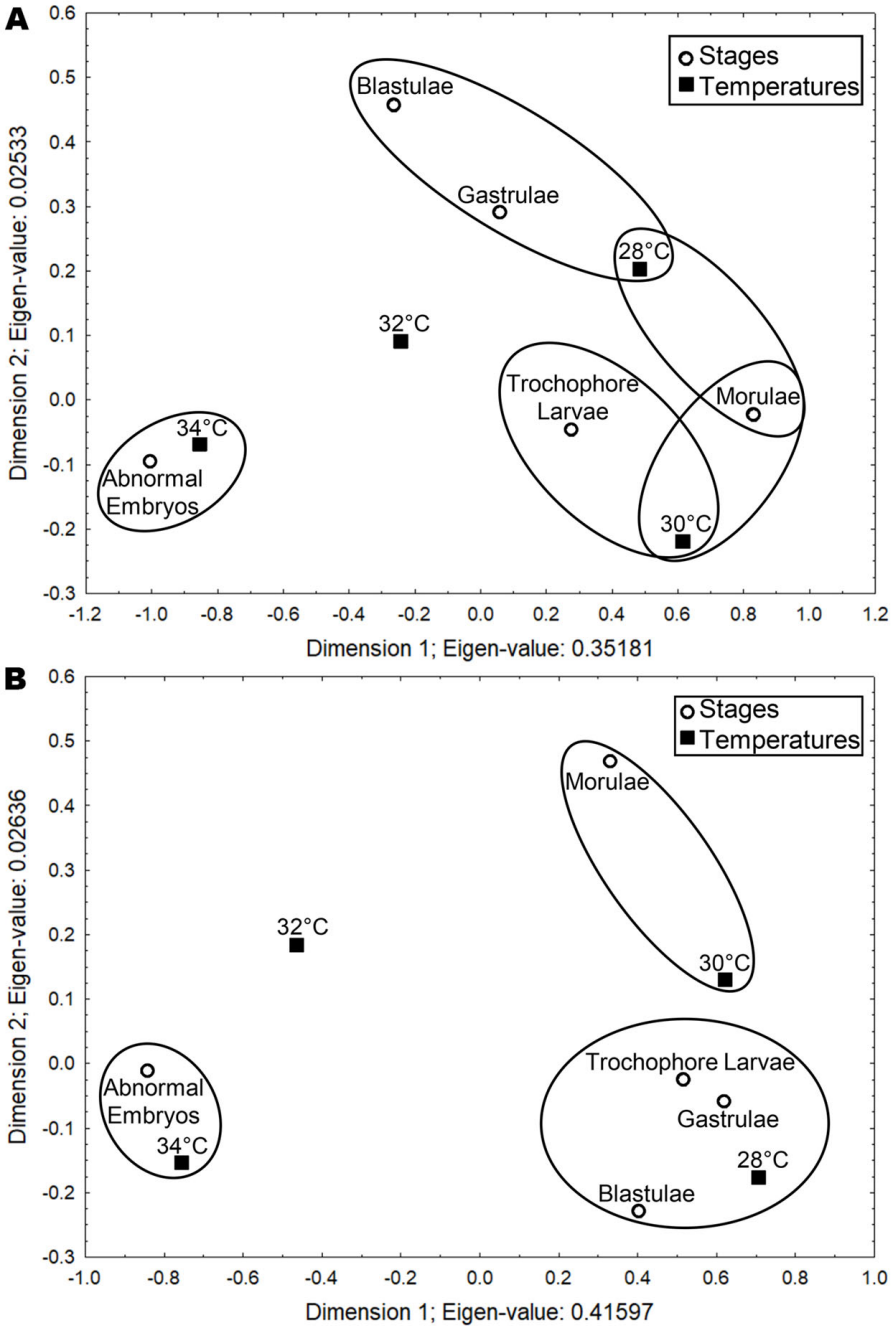
**Perceptual graphs of the Correspondence Analysis.** (A,B) Perceptual graph for *S. incrassatus* and *S.* cf. *corniculatus*, respectively, showing the correspondence between the temperature and developmental stages.

Correspondence Analysis for *S. incrassatus* indicated that the morulae had high correspondence with the temperatures of 28 and 30°C; the blastulae and gastrulae had high correspondence with 28°C, the trochophore larvae had high correspondence with 30°C, and abnormal embryos had high correspondence with 34°C (Total Inertia Index=0.37, Ha is accepted [X^2^_c=_156.64>X^2^_0.05_=21.02 (d.f.=12), *P*<0.05]) (Table 3, Fig. 5A). In *S.* cf. *corniculatus*, the morulae had high correspondence with 30°C, the blastulae, gastrulae and larvae had high correspondence with 28°C, and the abnormal embryos had high correspondence with 34°C (Total Inertia Index=0.44, Ha is accepted [X^2^_c_=167.95>X^2^_0.05_=21.02 (d.f.=12), *P*<0.05]) (Table 3, Fig. 5B). That is, the optimal temperatures for embryonic development of *S. incrassatus* and *S.* cf. *corniculatus* were 30 and 28°C, respectively. For both species, the maximum critical temperature was 32°C, in which all the stages from morulae to trochophore larvae were present, but in a lower percentage, and the percentage of abnormal embryos increased. The temperature of 34°C was the lethal temperature, in which the percentages of morulae to trochophore larvae decreased significantly and the percentage of abnormal embryos increased significantly.

**Table 3.**
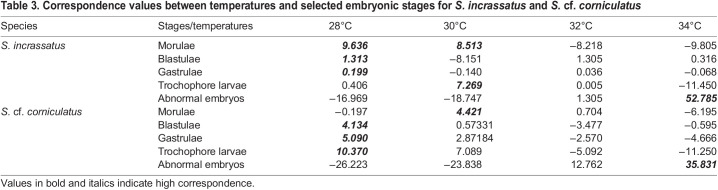
Correspondence values between temperatures and selected embryonic stages for *S. incrassatus* and *S.* cf. *corniculatus*

### Effect of temperature on the larval survival of *S. incrassatus* and *S.* cf. *corniculatus*

For *S. incrassatus*, the highest percentage of survival occurred in larvae exposed at 30°C [76.2±2.8%, (mean±standard deviation)], followed by those exposed at 28°C (70.5±4.5%) and 32°C (50.7±4.2%). The lowest percentage (28.9±2.6%) was observed at 34°C. Likewise, for *S.* cf. *corniculatus*, the highest percentage of larval survival occurred at 28°C (72.6±4.2%), followed by 30°C (57.6±1.1%) and 32°C (47.4±3.7%), and the lowest percentage was observed at 34°C (26.3±2.9%).

**Fig. 6.**
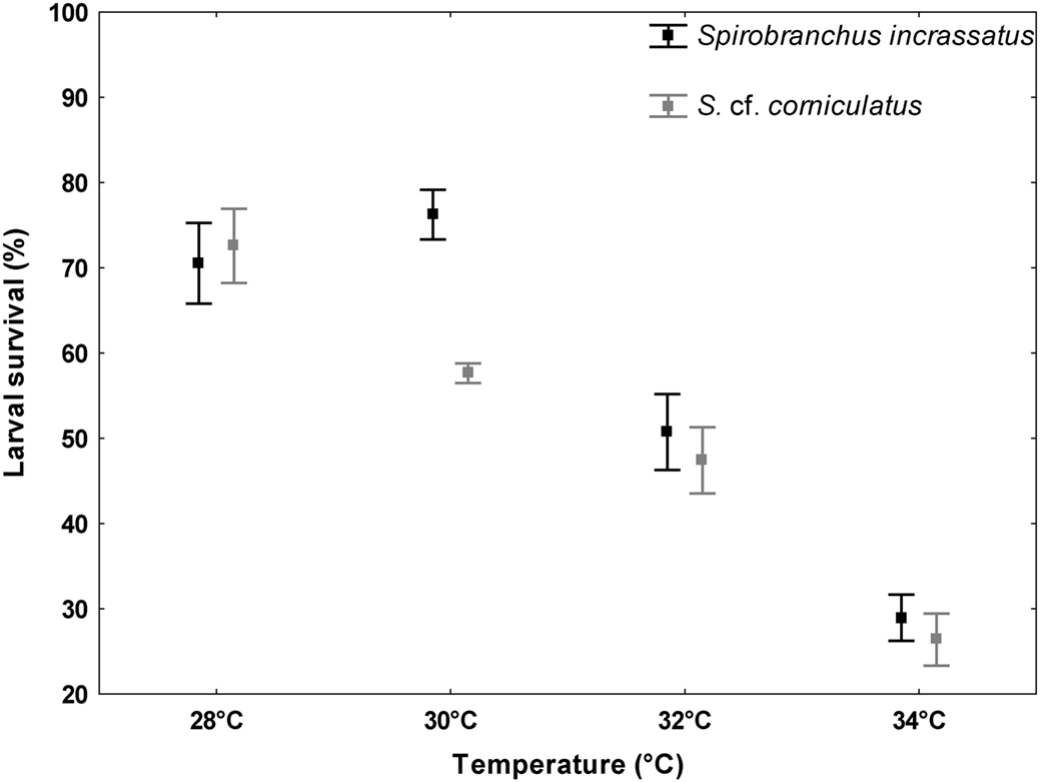
**Factorial analysis of variance (ANOVA).** Percentage of survival of larvae of *S. incrassatus* and *S.* cf. *corniculatus* at the different temperature treatments. The vertical lines denote 95% confidence intervals, and the squares indicate the means of the percentages of larval survival for both species (*n*=6).

The factorial ANOVA test showed that there were no statistically significant differences in the larval survival between experiments (*F*_(1, 32)_=3.13, *P=*0.086), but there were significant differences between species (*F*_(1, 32)_=40.15, *P<*0.001) and between temperatures (*F*_(3, 32)_=506.73, *P<*0.001). The Tukey HSD post-hoc test indicated that for *S. incrassatus* these differences were in all temperature combinations, except for 28 and 30°C, while for *S.* cf. *corniculatus*, there were differences in all temperature combinations. The comparison between both species indicated that there were only differences at 30°C (Fig. 6, Table 4).

**Table 4.**
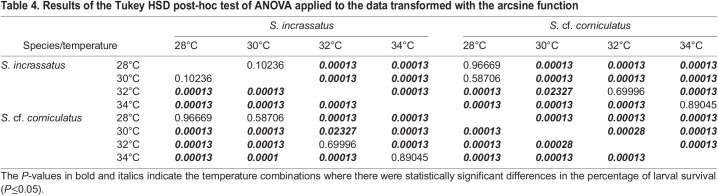
Results of the Tukey HSD post-hoc test of ANOVA applied to the data transformed with the arcsine function

## DISCUSSION

Numerous studies have indicated that temperature controls many stages of the life cycle of marine invertebrates and that increases in temperature often accelerate development. In ectothermic animals, such as *Spirobranchus* species, development is faster at warmer temperatures (Marsden, 1960; Crisp, 1977; Lacalli, 1977, 1981; Castric-Fey, 1984) because metabolism is accelerated at warmer temperatures (Gillooly et al., 2001, 2002). In the present study, the embryos of both species developed into larvae at a faster rate at 30°C.

In other species of the genus *Spirobranchus*, negative effects have also been reported due to the increase in temperature. At 30°C, embryos of *S. sinuspersicus* (before as *S. kraussii*) developed into larvae, but the larvae did not settle (Crisp, 1977), while at 34°C the lowest success of fertilization occurred the embryonic stage did not develop and cells denatured (Lavajoo and Amrollahi-Biuki, 2015). Gosselin et al. (2019) found that the population of *S. cariniferus* (Gray) from the west coast of New Zealand produced larger eggs and larvae that developed faster than the population of this same species inhabiting the east coast (the east coast is up to 2°C warmer than the west coast). It was concluded that this species is adapting to living in places with colder waters, thus facing global warming scenarios. Also, Sivananthan et al. (2021) indicated that fertilization and embryonic development of *S. bakau* can occur under a wide range of temperatures (25-31°C). Like these species, *S. incrassatus* and *S.* cf. *corniculatus* were negatively affected by the increase in temperature, that if the increase is too high (32 or 34°C), deleterious effects are observed on embryonic development and larval survival, although in the second species, these effects are observed from 30°C.

The intertidal species *S. incrassatus* showed a higher tolerance to temperature increase than the subtidal species *S.* cf. *corniculatus*. This may be because the maturing gametes of intertidal species are continually exposed to a greater temperature range than those of the subtidal species. As a result, mature eggs of intertidal species may be pre-packaged with heat-shock proteins, which are maternal derived, and accounts for their higher thermal tolerance (Hofmann, 1999; Clusella-Trullas et al., 2014).

The IPCC (2022) indicated that by 2100, the average temperature of the tropical seas, including the study area, could be 30 or 32°C, which means that under such conditions, there could be variations that would produce temperatures higher than 32°C. Like other tropical marine invertebrates, these two *Spirobranchus* species live close to their upper thermal tolerance limits (Somero, 2010, 2012). Therefore, in a global warming scenario, temperature increases will negatively affect several stages of the life cycle and, most likely, the distribution of both species in the study region. Future ocean warming could threaten the permanence of both species in the eastern tropical Pacific or at least lead to a contraction or fragmentation of their geographic distribution. It is important to mention that the populations studied in this study live in the warmest areas of their distribution range. Therefore, if sea temperature increases significantly, it could cause the disappearance of these populations that are living at the limit of their thermal tolerance. For other populations located at more temperate latitudes, favorable conditions could be generated for fertilization and survival of embryos and larvae. The disappearance of the studied populations would mean changes in the marine environment, leading to a loss of biodiversity in the study region. *Spirobranchus* species play a key role in marine environments because their calcareous tubes provide substrates for the settlement of other species of marine invertebrate, serving as habitat and refuge (Gosselin and Sewell, 2012; Pazoki et al., 2020; Sivananthan et al., 2021; Nishi et al., 2022). Additionally, there could be changes in the food chain, because serpulids are generally prey to many species of fish, echinoderms, and crustaceans. The disappearance of these species could also lead to changes in the water column. By feeding on suspended particles in the water column, serpulids contribute to the health of benthic ecosystems because they recycle nutrients from the suspended organic matter (Bastida-Zavala and Sánchez-Ovando, 2021).

On the Mexican Pacific coast, the effect of temperature on early development had not been evaluated in polychaetes. This type of information is very scarce for this study area and is only known for two species of sea urchins: *Strongylocentrotus purpuratus* (Stimpson) and *Toxopneustes roseus* (A. Agassiz). In precompetent and competent larvae of *S. purpuratus* lethal effects were produced, with 100% mortality (Díaz-Pérez and Carpizo-Ituarte, 2011). Mejía-Gutiérrez et al. (2019) observed that continuous exposure to a temperature of 32°C turned out to be deleterious for *T. roseus* with more than 90% abnormal embryos. When comparing the results of these authors with those obtained in this study, it was noted that the two species of sea urchins were more vulnerable to the negative effects of the increase in temperature than the two species of polychaetes studied here.

The results of our study suggest that *S.* cf. *corniculatus* is the species with the lowest thermal tolerance and the two *Spirobranchus* species already live near their upper limit of thermal tolerance in the study region (30°C). Our study provides an initial view of the possible negative effects on early development due to temperature increases predicted for the near future.

## MATERIALS AND METHODS

### Study area

The specimens of *S. incrassatus* were collected on the pier pillars in the Puerto Ángel Harbor (15°40′18″ N, 96°29′45″ W), in the intertidal zone at 0.5-1 m; while specimens of *S.* cf. *corniculatus* were collected on rocky areas in the Estacahuite Bay (15°40′5.15″ N, 96°28′51.94″ W), in the subtidal zone at 5-6 m (Fig. 1). Both locations belong to the western margin of the Gulf of Tehuantepec, Mexico.

### Field work

Samples were collected monthly between May and December 2019; however, in July we did not collect specimens due to heavy rains. Collections were made by SCUBA diving during low tide. A total of 11 sampling events were performed, seven on the pier pillars in Puerto Ángel Harbor and four at Estacahuite Bay.

During each sampling, adult worms within their calcareous tubes were removed from the substratum using a hammer and chisel. Immediately after, they were transported in plastic bags to the Laboratorio de Ecología del Desarrollo (ECODES, Universidad del Mar, Oaxaca, Mexico). Adults were maintained for 3-4 h under controlled conditions, with constant aeration, salinity 35, temperature ∼28°C and pH 8.1.

After the experiment, all *Spirobranchus* specimens were maintained in the Polychaete Section of the Scientific Collection (OAX-CC-249-11) of the Laboratorio de Sistemática de Invertebrados Marinos (LABSIM), Universidad del Mar, Oaxaca, Mexico.

### Laboratory work

#### Experimental design and spawning

Four water baths (Grant W14 model, Grant Instruments, Cambridge, UK) were used, which were filled with 10 l of distilled water and set to four temperatures (28, 30, 32, and 34°C). The experiments with embryos and larvae were carried out three times per sampling date for a total of six replicates from the pooled experiment 1 (E1) and experiment 2 (E2). A temperature of 28°C was considered as the control, as it is the historical average seawater temperature in the study area. A temperature of 30°C was considered as the summer temperature, when the most intense reproductive activity had been observed for several marine invertebrates in the study area (Avila-Poveda, 2013; Benítez-Villalobos and Martínez-García, 2012; Benítez-Villalobos et al., 2013, 2015). A temperature of 32°C (an increase of 4°C above the average) was considered as the most extreme scenario predicted by the IPCC for the region by 2100, where the average sea temperature is expected to increase by 1.6-4°C, depending on the geographic region (Brierley and Kingsford, 2009; Meinshausen et al., 2011; IPCC, 2022). A temperature of 34°C represents a catastrophic warming scenario, which has been tested in other tropical marine invertebrates (Rahman et al., 2009; Sarifudin et al., 2016); therefore, it was also used to determine its effect on these *Spirobranchus* species.

The ambient temperature of the laboratory was maintained at 20°C to avoid fluctuations in the temperature of each bath. The seawater used in the experiment was mechanically filtered with a 1 µm mesh and irradiated with UV light. Two beakers were filled with 300 ml of filtered seawater (FSW), sealed with plastic wrap, and placed inside each water bath. These procedures were completed a day before experimental treatments to maintain a stable temperature in each water bath.

Two experiments for each species of *Spirobranchus* were completed (*S. incrassatus*: August 30th and December 4th, 2019; *S.* cf. *corniculatus*: November 7th and 21st, 2019) using pooled gametes from different parents (25 adult females and five adult males per species). Before the spawning induction, the organisms were washed with FSW to eliminate epibionts and the remains of organic matter that had adhered to their tubes. Spawning was induced by removing 30 adult worms from their tubes without damaging the delicate body of the worms. Usually, when the serpulids are removed from their tubes, they begin to release their gametes. The males were put in small containers without water to obtain sperm and females were left to release eggs into the FSW. The gametes were collected using a micropipette and examined under microscope to confirm their viability (sperm motility and breakdown of germinal vesicle in eggs).

#### Effect of temperature on embryonic development

Once the gametes were obtained and tested for viability, the eggs were placed in a sieve of 55 µm mesh, where they were cleaned by gently washing with FSW, then placed in a 2.3 l plastic jar with FSW. Then, 1 ml of concentrated sperm was added to the jar. The gamete mixture was gently stirred and left to rest for 15-30 min, thus achieving fertilization. The zygotes were placed in a sieve of 55 µm mesh and washed to remove excess sperm. The clean zygotes were divided into four portions of 300 ml each (one for each temperature treatment) and placed in the beakers with FSW that were at each temperature. Subsequently, counts were made at each beaker and a final suspension of 20 zygotes ml^−1^ was obtained in each one. The remaining embryos in the plastic jar developed into trochophore larvae 12-14 h post-fertilization to be used later in the larval survival experiments.

Eighteen glass vials were placed at each temperature treatment (28, 30, 32, and 34°C) and each vial was filled with 15 ml of the zygote suspension corresponding to each temperature treatment. The suspension of all the vials was oxygenated with the help of an air pump. Three vials were extracted for each temperature every 4 h and the content of each vial was fixed with 1 ml of 37% formalin until completing 24 h. In each vial 100 embryos were counted with a Sedgewick-Rafter chamber, identifying the stages: morulae, blastulae, gastrulae until early trochophore larvae (as described in Sánchez-Ovando et al., 2021), as well as abnormal embryos; thus, recording the percentage of each one. We considered abnormal embryos as those embryos that had blastomeres with alterations in size, as well as broken egg envelopes.

#### Effect of temperature on larval survival

The larval culture in the plastic jar was cleaned by gently washing with FSW and then divided into four portions (one for each temperature treatment) with a larval density of 15 larvae ml^−1^ per portion.

Three glass vials were placed at each temperature treatment (28, 30, 32, and 34°C) and each vial was filled with 15 ml of the larvae suspension corresponding to each temperature treatment. The suspension of larvae of all the vials was oxygenated with the help of an air pump. The vials for each treatment were left for 24 h in water baths, after which they were extracted. Subsequently, with the help of a microscope and a Bogorov chamber, 100 larvae were counted in each vial, and if they were alive or dead was recorded. This allowed us to determine the average percentage of larval survival at each temperature.

It should be noted that in this work only the survival of early larvae exposed to temperature treatments for 24 h was examined. That is, the experiment ended when the larvae were already 36-38 h post-fertilization, before the larvae needed to be fed.

### Statistical analysis

#### Embryonic development

For each species, the data obtained from experiments 1 (E1) and 2 (E2) were grouped into a data matrix, and the average percentage of embryos was obtained at each sampling time to create bar graphs. Since these data did not meet the assumptions of normality and homoscedasticity, we decided to use a nonparametric test, the Analysis of Similarities (ANOSIM). The ANOSIM was used to determine statistically significant differences in the percentage of embryos of each stage between and among temperatures and between and among E1 and E2. Differences were considered to be statistically significant when *P*≤0.05.

A contingency matrix was structured to apply a Correspondence Analysis (CA) using STATISTICA© version 7.0 software. The CA was used to evaluate the possible correspondence between the stages (abnormal embryos, morulae, blastulae, gastrulae and trochophore larvae) and the temperature treatments (28, 30, 32 and 34°C). This allowed us to identify the optimal and lethal temperatures and to explain in terms of ‘affectation’, how the increase in temperature affected five of the embryonic stages of *S. incrassatus* and *S.* cf. *corniculatus*.

#### Larval survival

The data matrix of experiments 1 (E1) and 2 (E2) of both species was integrated into a single matrix. This matrix was analyzed with an ANOVA using STATISTICA. The factorial ANOVA was used to determine significant differences in the percentage of larval survival between three factors: experiments (E1 and E2), species (*S. incrassatus* and *S.* cf. *corniculatus*), and temperature treatments (28, 30, 32 and 34°C). In addition, a Tukey HSD post-hoc test was used to determine which temperature combinations had significant differences. Differences were considered to be statistically significant when *P*≤0.05. The data being percentages were transformed with the arcsine function and fulfilled the assumptions of normality and homoscedasticity (Zar, 2010). These assumptions were verified with the Shapiro-Wilks (*P*>0.05) and Levene (*P*>0.05) tests, respectively.
